# The New York Standard

**Published:** 1904-09

**Authors:** 


### "The New York Standard."

UNDER this title the *Columbus Medical Journal*, in its August issue, discusses the methods in vogue in this state with reference to the examinations for state medical licensure with a candor and intelligence that is most gratifying. Especially is this the case when we recall the many spasmodic, if not hysterical, attacks made upon the New York method, under cover of the "reciprocity" special pleadings, by the sophomoric editors in the middle and remoter west, who display meager knowledge of the subject. We take pleasure in reproducing the article in question in its full text:

The standard exacted by the regents of New York for that state is that no college shall be recognised by the board which does not make four years of actual attendance upon medical lectures a prerequisite to graduation for all students. Colleges, therefore, which give advance standing to graduates of literary colleges will not be recognised in that state and their graduates even those who have taken the full four years' course, will not be permitted to the examinations. So far as appears in their several announcements, the Ohio Medical University is the only one of the Ohio colleges that has thus far adopted the new standard. It is to be hoped that other colleges of the state will soon make the same requirement, and that the State Board of Medical Registration and Examination will adopt this four years' standard. The standard is none too high, but the advance must be made all along the line. In a state like Ohio with medical colleges so generally distinct from literary colleges it makes the two courses cover eight years, but steps could soon be taken to provide for the seven years' complete course. The standard set by New York, if maintained, will no doubt be followed by other states, and will be far-reaching in its influence. It is not the intention of the new standard to ignore the advantages of literary education, but rather to maintain the integrity of the four years' medical course. Hitherto, pride in the classic literary course with many of the older institutions, shut out electives, and made it cover four full years, and then, providing certain sciences entered into this literary course, it has hitherto been assumed that the medical course could be completed in three years. But it is found that students with advanced standing labor under disadvantages, and have not been able to do the work well. Anatomy, physiology, bacteriology and histology, which are rarely—it may be said are never—taught in literary (distinct from medical) colleges, and the other three years are crowded full of medical studies, chemistry being about the only study which has been well mastered in the literary course. The loss of a year's work in these important fundamental branches has been found to seriously handicap the brightest minds; they will lack, notwithstanding their superior literary education, that ready grasp and insight, that poise and self-confidence which characterise the student who is well grounded in first year's anatomy, chemistry, physiology, histology and bacteriology. Therefore the New York State Board has decided to recognise no college that does not maintain the full four years' course for all students, giving advanced standing to none, credit being given, however, for all work actually done.

One result of this action will be to bring literary and medical colleges closer together; the former will probably find that they can place first year medical studies as electives late in a literary course for those who desire to begin the study of medicine and permit their students to elect medical studies for their last year and pursue them in some accredited medical college, thus enabling students in Ohio, where we have so many independent colleges, to complete the two courses in seven years, receiving the bachelor's degree from the literary college at the end of the first year's medical course. This would create a bond of interest between the literary and medical colleges which would in time doubtless lead to the mergement of the latter into favorably located literary universities, a decided gain for the cause of education.

The Ohio State Board can greatly promote the cause of medical education and elevate the profession in Ohio by adopting a like requirement. It would place a premium upon thorough preparation for the medical profession, preserve the integrity of the medical course, and make it easier to raise the entrance requirements for those who were not college graduates. The members of the profession should interest themselves in this matter and urge upon the state board that this advanced step be taken at an early date.

